# Supplementary material for: Allelopathic Toxicity of Cyanamide Could Control Amaranth (Amaranthus retroflexus L.) in Alfalfa (Medicago sativa L.) Field
Source: Molecules. 2022 Oct 28;27(21):7347. doi: 10.3390/molecules27217347 (PMC9658055; doi:10.3390/molecules27217347)
Supplement: Supplementary file 1 [file molecules-27-07347-s001.zip › molecules-1971042-supplementary.pdf]

Table S1. The non-linear fit of seed germination process.

		Non-Linear Fitting	R^2	F	*P*	GR50
Amaranthus retroflexus	RI of germination rate	$y=2006.015x^2-1060.618\times + 7.644$	0.893	16.751	0.011	0.062
	RI of radicle length	$y=14079.905x^2-2400.719x+7.211$	0.944	17.010	0.056	0.029
	RI of hypocotyl length	$y=1181.438x^2-487.067x-62.248$	0.931	13.542	0.069	-
	RI of fresh weight	$y=-1489.768x^2-611.466x-23.816$	0.968	30.117	0.032	0.039
	RI of dry weight	$y=-5789.76x^2+259.042x-68.98$	0.680	2.126	0.32	0.034
	SE	$y=5214.15x^2-1352.003x-15.064$	0.999	736.203	0.001	0.029
Medicago sativa	RI of germination rate	$y=-1008.727x^2+180.951x-7.630$	0.981	105.085	0.000	0.313
	RI of radicle length	$y=-10244.184x^2+1064.2x-18.243$	0.341	0.517	0.659	0.142
	RI of hypocotyl length	$y=-3489.447x^2+284.58x-11.665$	0.392	0.644	0.608	0.153
	RI of fresh weight	$y=-573.764x^2-188.25x+13.192$	0.725	2.637	0.275	0.206
	RI of dry weight	$y=1480.904x^2-231.076x+1.075$	0.967	2.007	0.033	-
	SE	$y=-437.531x^2-47.349x-3.205$	0.782	3.591	0.218	0.277

Table S2. The non-linear fit of seedling growth process.

		Regression Analysis	R^2	F	*P*	GR50 Value
Amaranthus retroflexus	RI of root length	$y=-54.087x^2+68.339x-41.054$	0.977	42.721	0.023	1.383
	RI of stem length	$y=23.563x^2-81.427x+18.069$	0.993	144.308	0.007	1.417
	RI of fresh weight	$y=25.474x^2-77.086x-20.743$	0.715	2.514	0.285	0.445
	RI of dry weight	$y=39.232x^2-108.435x+1.134$	0.924	12.228	0.076	0.603
	SE	$y=8.537x^2-49.638x-10.656$	0.956	21.808	0.044	0.945
Medicago sativa	RI of root length	$y=-39.360x^2+47.567x-12.142$	0.976	40.586	0.024	1.757
	RI of stem length	$y=-51.019x^2+81.204x-34$	0.989	89.643	0.011	1.769
	RI of fresh weight	$y=-43.535x^2+69.228x-51.995$	0.248	0.329	0.752	1.561
	RI of dry weight	$y=-59.454x^2+71.04x-20.993$	0.98	49.06	0.02	1.517
	SE	$y=-48.342x^2+67.26x-29.782$	0.902	9.201	0.098	1.646
